# Genomic Characterization of SARS-CoV-2 Isolated from Patients with Distinct Disease Outcomes in Mexico

**DOI:** 10.1128/spectrum.01249-21

**Published:** 2022-01-12

**Authors:** Celia Boukadida, Blanca Taboada, Marina Escalera-Zamudio, Pavel Isa, José Ernesto Ramírez-González, Joel Armando Vazquez-Perez, José Esteban Muñoz-Medina, Concepción Grajales-Muñiz, Carolina González-Torres, Francisco Javier Gaytán-Cervantes, Alma Rincón-Rubio, Margarita Matías-Florentino, Héctor Esteban Paz-Juárez, Alejandro Sanchez-Flores, Edgar Mendieta-Condado, Gisela Barrera-Badillo, Lucía Hernández-Rivas, Susana López, Irma López-Martínez, Santiago Ávila-Ríos, Carlos F. Arias

**Affiliations:** a Centro de Investigación en Enfermedades Infecciosas, Instituto Nacional de Enfermedades Respiratorias Ismael Cosío Villegas, Mexico City, Mexico; b Departamento de Genética del Desarrollo y Fisiología Molecular, Instituto de Biotecnología, Universidad Nacional Autónoma de México, Cuernavaca, Morelos, Mexico; c Department of Zoology, Oxford University, Oxford, United Kingdom; d Instituto de Diagnóstico y Referencia Epidemiológicos, Dirección General de Epidemiología, Mexico City, Mexico; e Instituto Nacional de Enfermedades Respiratorias Ismael Cosío Villegas, Mexico City, Mexico; f División de Laboratorios de Vigilancia e Investigación Epidemiológica, Instituto Mexicano del Seguro Social, Mexico City, Mexico; g Coordinación de Control Técnico de Insumos, Instituto Mexicano del Seguro Social, Mexico City, Mexico; h División de Desarrollo de la Investigación, Instituto Mexicano del Seguro Social, Mexico City, Mexico; i Unidad Universitaria de Secuenciación Masiva y Bioinformática, Instituto de Biotecnología, Universidad Nacional Autónoma de México, Cuernavaca, Morelos, Mexico; The Ohio State University

**Keywords:** SARS-CoV-2, genomic diversity, Mexico, distinct clinical outcomes

## Abstract

The coronavirus disease 2019 (COVID-19), caused by the severe acute respiratory syndrome coronavirus-2 (SARS-CoV-2), has shown a wide spectrum of clinical manifestations ranging from asymptomatic infections to severe disease and death. Pre-existing medical conditions and age have been mainly linked to the development of severe disease; however, the potential association of viral genetic characteristics with different clinical conditions remains unclear. SARS-CoV-2 variants with increased transmissibility were detected early in the pandemics, and several variants with potential relevance for public health are currently circulating around the world. In this study, we characterized 57 complete SARS-CoV-2 genomes during the exponential growth phase of the early epidemiological curve in Mexico, in April 2020. Patients were categorized under distinct disease severity outcomes: mild disease or ambulatory care, severe disease or hospitalized, and deceased. To reduce bias related to risk factors, the patients were less than 60 years old and with no diagnosed comorbidities A trait-association phylogenomic approach was used to explore genotype–phenotype associations, represented by the co-occurrence of mutations, disease severity outcome categories, and clusters of Mexican sequences. Phylogenetic results revealed a higher genomic diversity compared to the initial viruses detected during the early stage of the local epidemic. We identified a total of 90 single nucleotide variants compared to the Wuhan-Hu-1 genome, including 54 nonsynonymous mutations. We did not find evidence for the co-occurrence of mutations associated with specific disease outcomes. Therefore, in the group of patients studied, disease severity was likely mainly driven by the host genetic background and other demographic factors.

**IMPORTANCE** The genetic association of severe acute respiratory syndrome coronavirus-2 (SARS-CoV-2) with different clinical conditions remains unclear and needs further investigation. In this study, we characterized 57 complete SARS-CoV-2 genomes from patients in Mexico with distinct disease severity outcomes: mild disease or ambulatory care, severe disease or hospitalized, and deceased. To reduce bias related to risk factors the patients were less than 60 years old and with no diagnosed comorbidities. We did not find evidence for the co-occurrence of mutations associated with specific disease outcomes. Therefore, in the group of patients studied, disease severity was likely mainly driven by the host genetic background and other demographic factors.

## INTRODUCTION

The coronavirus disease 2019 (COVID-19), declared a pandemic by the WHO on March 11th 2020, is caused by a coronavirus of zoonotic origin that was recently introduced into the human population, named the severe acute respiratory syndrome coronavirus 2 (SARS-CoV-2) (1). SARS-CoV-2, first detected in December 2019, belongs to the *Sarbecovirus* subgenus, *Betacoronavirus* genus of the *Coronaviridae* family (1), together with SARS-CoV-1, which caused a short-lived outbreak of great medical importance in 2002 (2). As of October 2021, the COVID-19 pandemic had caused over 240 million cases and 4.9 million deaths globally, as reported by the World Health Organization (3). Genomic epidemiology and surveillance efforts worldwide have rendered more than 2.3 million full SARS-CoV-2 genome sequences available in public data repositories such as the Global Initiative on Sharing Avian Influenza Data (GISAID) platform (4, 5). Studying virus genome sequences in an evolutionary context has allowed to explore in real time the impact of genetic changes occurring in the circulating virus populations within different geographical regions (6–8). As a result, an increasing number of SARS-CoV-2 variants displaying different mutations have become of potential relevance for public health (9). Some of these mutations encode amino acid changes within the spike (S) protein of the virus, which mediates its attachment to the host cell receptor through its globular domain and is the main target for the neutralizing antibody response (10). Some of these variants seem to be associated with immune escape or increased transmissibility (11, 12).

As an example, the SARS-CoV-2 variant displaying the amino acid change D614G in the S protein emerged during late January to early February 2020 and became predominant on a global scale during March–April 2020. The mutation D614G is associated with increased transmissibility of the virus (13–16). At the molecular level, the D614G change was demonstrated to favor a slightly more open conformation of the spike protein with added interactions that prevent premature dissociation of the trimer and modulate structural rearrangements for membrane fusion, effectively enhancing infectivity (17, 18). Comparably, recent epidemiological reports suggest that viral variants belonging to the B.1.1.7 (Alpha), B.1.351 (Beta), P.1 (Gamma), and B.1.617.2 (Delta) expanding lineages could also be associated with an increased transmissibility and immune escape (19). Such variants are currently classified as variants of concern (VOC) (20), and share amino acid changes in the S protein that in some cases have emerged in parallel, such as the K417N/T (in B.1.351 and P.1), N501Y (in B.1.1.7, B.1.351, and P.1), E484K (in B.1.351 and P.1) or P681H/R (in B.1.1.7 and B.1.617.2) (9). The N501Y substitution introduces an additional interaction with the angiotensin converting enzyme 2 (ACE2) receptor, increasing the binding affinity, and abolishes the binding of a potent neutralizing antibody (21, 22). The E484K change was also demonstrated to increase the binding affinity of the spike receptor binding domain (RBD) for ACE2 (23). In addition, E484K confers resistance to several monoclonal antibodies and significantly reduces neutralization by convalescent plasma and sera obtained from vaccinated individuals, indicating that this substitution is located in an immunodominant epitope (11, 19, 24).

A wide spectrum of clinical manifestations have been observed in symptomatic SARS-CoV-2 infected individuals, ranging from asymptomatic infections or mild upper-respiratory tract symptoms treated under ambulatory care, to severe pneumonia and multiorgan failure, resulting in hospitalization and treatment within the intensive care unit that may ultimately lead to death (25). Demographic characteristics such as age and sex have been linked to the development of a severe disease and/or death in the context of COVID-19 (26). Additional identified risk factors are associated with underlying medical conditions such as obesity, cancer, cardiovascular disease, diabetes, immunosuppression, as well as other respiratory, kidney, liver and neurological conditions (26–30).

The potential role of viral mutations in disease severity remains unclear, and to date, there is insufficient evidence supporting that the genetic background of SARS-CoV-2 may play a role in disease outcome (31, 32). However, as the genetic diversity of the virus increases, exploring the potential impact of emerging mutations related to virulence in the human host (i.e., by increasing transmissibility, or linked to a worse disease outcomes) remains an important task. In this study, we characterized 57 complete SARS-CoV-2 genomes generated from Mexican samples derived from patients categorized under distinct disease severity outcomes: *a*mbulatory care or mild disease (A); *h*ospitalized or severe disease (H); and *d*eceased (D). To reduce bias related to risk factors in the sampled population, patient inclusion criteria were limited to less than 60 years of age and no diagnosed comorbidities. A trait-association phylogenomic approach was used to explore genotype–phenotype associations, represented by the co-occurrence of mutations, disease severity outcome categories, and clusters of Mexican sequences.

## RESULTS

### Clinical sample distribution.

The Mexican isolates studied here were collected during the exponential growth phase of the early epidemiological curve in Mexico, from 03/26/2020 to 04/30/202, (Table S1, Fig. S1 in the supplemental material). A total of 57 genomic sequences were obtained, and for their analysis the patients were categorized into three groups according to disease severity, based on the clinical history and the official disease outcome recorded for each patient: *a*mbulatory care (A, *n* = 24), severe disease or *h*ospitalized (H, *n* = 14), and fatal or *d*eceased (D, *n* = 19) (Table 1). The samples were selected from those patients who met the inclusion criteria at the time of the study; the median age of the patients was 49 years (range 22 to 59) (Table S3).

The samples were obtained from 7 out of 32 states in the country, with those from Mexico City being predominant (Table 1). This biased pattern is likely to reflect either a higher number of reported cases in the capital city or the geographical uneven stage of the epidemic in the country (33) (Fig. S1). Differences in genomic surveillance efforts at a regional level, with most surveillance occurring in Mexico City, are also expected to influence sample distribution. Significant differences in patient age and the sites where the sample were collected were observed across the three severity groups (A, H, and D). Sex was the only category with no significant difference observed across groups (Table 1). On average, patients were significantly younger within the group “A”, compared to the “D” and “H” groups, while samples from Mexico City were overrepresented within the “H” and “D” groups.

**TABLE 1:** Characteristics of the study participants^*a*^

Disease outcome category	Ambulatory care (A)	Hospitalized (H)	Deceased (D)	*P* values
	*n* = 24	*n* = 14	*n* = 19	
Age (yrs), median (range)	34 (24-50)	47 (22-59)	45 (27-59)	** P = 0.0013
Sex (Male), *n* (%)	15 (62.5%)	10 (71.4%)	15 (78.9%)	NS, *P = 0.5004*
Location				
Mexico City, *n* (%)	8 (33.3%)	11 (78.6%)	11 (57.9%)	*
Other states of Mexico, *n* (%)	16 (66.7%)	3 (21.4%)	8 (42.1%)	***P = *0.0227**
Symptoms Fever, *n* (%) Cough, *n* (%) Headache, *n* (%) Sore throat, *n* (%) Fatigue, *n* (%) Myalgia, *n* (%) Arthralgia, *n* (%) Rhinorrhea, *n* (%) Conjunctivitis, *n* (%) Dyspnea, *n* (%) Diarrhea, *n* (%) Chest pain, *n* (%) NA, *n*	20 (83.3%) 21 (87.5%) 22 (91.7%) 15 (62.5%) 16 (66.7%) 15 (62.5%) 16 (66.7%) 12 (50.0%) 3 (12.5%) 1 (4.2%) 2 (8.3%) 8 (33.3%) 0	11 (78.6%) 10 (71.4%) 7 (50.0%) 6 (42.9%) 7 (50.0%) 7 (50.0%) 8 (57.1%) 6 (42.9%) 1 (7.1%) 12 (85.7%) 5 (35.7%) 3 (21.4%) 0	16 (88.9%) 17 (94.4%) 8 (44.4%) 4 (22.2%) 9 (50.0%) 10 (55.6%) 11 (61.1%) 4 (22.2%) 2 (11.1%) 17 (94.4%) 2 (11.1%) 6 (33.3%) 1	NS, *P* = 0.73 NS, *P* = 0.17 ** P = 0.0020 * P = 0.0338 NS, *P* = 0.46 NS, *P* = 0.74 NS, *P* = 0.83 NS, *P* = 0.18 NS, *P* = 0.87 **** P < 0.0001 NS, *P* = 0.07 NS, *P* = 0.70
Time from symptom onset to diagnosis (days), median (interquartile range)	2.5 (1–4)	8 (2.5–13.25)	6 (4.75–8.25)	** P = 0.0018

a Kruskal-Wallis and Chi-square tests were used to compare continuous and categorical variables, respectively (NS, not significant; *, *p* ≤ 0.05; **, *p* ≤ 0.005; ****, *p* < 0.0001). NA, not available. In bold, the values that were statistically significant.

Differences in the clinical presentation were also observed across the three severity groups. As expected, dyspnea was markedly more frequent in hospitalized and deceased patients compared to individuals with asymptomatic/mild disease (Table 1). In contrast, at the time of diagnosis, patients in group “A” more frequently presented headache and sore throat compared to the “H” and “D” groups. Since the time from symptom onset to diagnosis was significantly longer for individuals with severe and fatal outcomes, the observed lower frequency of headache and sore throat in these patients might reflect the resolution of early COVID-19 symptoms as described previously (34, 35). Other symptoms were found at similar frequencies in the three severity groups.

### Genomic and phylogenetic characterization of Mexican isolates.

**FIG 1:**
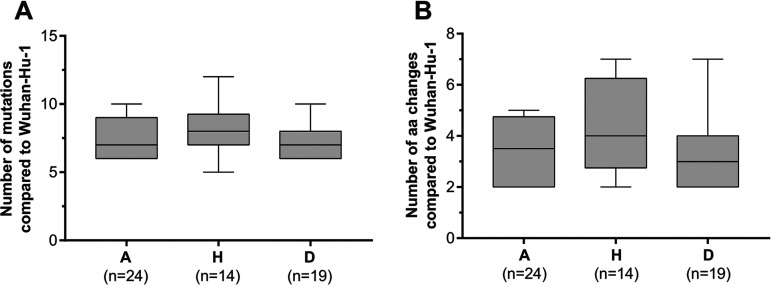
Genome variation compared to Wuhan-Hu-1 across severity groups. (A) Number of mutations and (B) amino acid changes, compared to the reference genome Wuhan-Hu-1 in the ambulatory care (A), hospitalized (H), and deceased (D) study groups. Boxes contain the 25th and 75th percentiles, and whiskers show the minimum and maximum values.

The 57 Mexican virus genome sequences displayed good quality, as shown by a median genome coverage of 99.9% (range: 98.5%–99.99%) and median depth of 1,274× (range: 33×–37,020×) (Table S1). The median pairwise genetic distance observed across the 57 genomes was of 6 nucleotides (range: 0–14), reflecting a low genetic diversity, consistent with observations made on viral diversity at a global scale during this period (36, 37). The Mexican sequences showed between 5 to 12 nucleotide and 2 to 7 amino acid changes compared to the reference genome (Fig. 1, Table 2, Table S1). No significant differences in the number of mutations or amino acid changes within the Mexican sequences were detected across the different groups (A, H, or D) (Fig. 1A and B, Kruskal-Wallis tests, *P* = 0.19 and *P* = 0.17 respectively).

**TABLE 2:** Description of full-length SARS-CoV-2 genomes^*a*^

Sample ID	Pango lineage	# of changes		aa changes
		nt^*b*^	aa^*b*^	
25_INER_49A_M_MEX_D_26/03/2020	B.1.609	6	2	Orf1b:P314L,S:D614G
42_INER_53A_M_CMX_D_30/03/2020	B.1.609	7	3	Orf1a:Q4189H,Orf1b:P314L,S:D614G
68_INER_40A_M_MEX_H_04/04/2020	B.1.609	8	2	Orf1b:P314L,S:D614G
75_INER_34A_M_CMX_A_04/04/2020	B.1.1.222	9	5	N:R203K,N:G204R,Orf1b:P314L,S:D614G,S:T732A
83_INER_39A_M_CMX_D_06/04/2020	B.1.609	7	3	Orf1b:P314L,S:A260S,S:D614G
85_INER_27A_M_CMX_A_06/04/2020	B.1	7	4	Orf1a:L3338F,Orf1b:P314L,Orf3a:Q57H,S:D614G
91_INER_45A_F_MEX_D_07/04/2020	B.1.609	6	2	Orf1b:P314L,S:D614G
95_INER_56A_M_CMX_D_13/04/2020	B.1.609	9	3	Orf1a:I1276T,Orf1b:P314L,S:D614G
104_INER_43A_M_CMX_D_10/04/2020	B.1.111	7	4	Orf1b:P314L,Orf3a:Q57H,Orf7a:T14I,S:D614G
106_INER_36A_F_CMX_H_10/04/2020	B.1	5	2	Orf1b:P314L,S:D614G
109_INER_41A_F_MEX_D_08/04/2020	B.1	6	2	Orf1b:P314L,S:D614G
112_INER_28A_M_CMX_A_08/04/2020	B.1	7	5	Orf1a:T265I,Orf1a:D3972E,Orf1b:P314L,Orf3a:Q57H,S:D614G
118_INER_59A_M_CMX_D_08/04/2020	B.1.609	7	2	Orf1b:P314L,S:D614G
126_INER_56A_M_MEX_D_11/04/2020	B.1.1	9	5	N:R203K,N:G204R,Orf1a:A1049V,Orf1b:P314L,S:D614G
137_INER_58A_M_CMX_D_13/04/2020	B.1.1	10	7	N:R203K,N:G204R,Orf1a:I404T,Orf1a:H712Y,Orf1b:A176V,Orf1b:P314L,S:D614G
173_INER_34A_M_CMX_H_15/04/2020	B.1.1	12	6	M:A69S,N:R203K,N:G204R,Orf1b:P314L,Orf1b:L1629S,S:D614G
192_INER_39A_M_CMX_D_17/04/2020	B.1.609	8	4	Orf1a:Y1920H,Orf1a:A4285V,Orf1b:P314L,S:D614G
204_INER_52A_M_CMX_H_18/04/2020	B.1	7	5	Orf1a:T265I,Orf1b:P314L,Orf3a:Q57H,S:D614G,S:D936Y
205_INER_34A_M_CMX_A_18/04/2020	B.1	7	3	Orf1b:P314L,Orf1b:V1768I,S:D614G
207_INER_36A_M_MEX_H_17/04/2020	B.1	9	7	Orf1a:K141R,Orf1a:T265I,Orf1b:P314L,Orf1b:G2197D,Orf3a:Q57H,S:D614G, S:D936Y
221_INER_59A_M_CMX_H_18/04/2020	B.1.609	7	2	Orf1b:P314L,S:D614G
228_INER_53A_F_CMX_H_19/04/2020	B.1	9	7	Orf1a:K141R,Orf1a:T265I,Orf1b:P314L,Orf1b:G2197D,Orf3a:Q57H,S:D614G, S:D936Y
229_INER_50A_M_CMX_H_19/04/2020	B.1.609	10	5	N:S327L,Orf1a:G3072C,Orf1b:P314L,Orf3a:S166L,S:D614G
238_INER_59A_M_CMX_D_21/04/2020	B.1.609	6	2	Orf1b:P314L,S:D614G
260_INER_43A_M_MEX_D_22/04/2020	B.1.609	7	2	Orf1b:P314L,S:D614G
275_INER_45A_M_CMX_D_25/04/2020	B.1	6	3	Orf1b:P314L,S:V143F,S:D614G
286_INER_48A_M_MEX_H_26/04/2020	B.1.609	8	4	Orf1a:A4285V,Orf1b:P314L,S:D614G,S:V1268I
296_INER_38A_F_CMX_H_23/04/2020	B.1	7	3	Orf1b:P314L,Orf3a:Q57H,S:D614G
317_INER_55A_F_CMX_H_27/04/2020	B.1	9	4	Orf1a:L1249F,Orf1a:N1995H,Orf1b:P314L,S:D614G
689_InDRE_38A_F_YUC_D_10/04/2020	B.1	6	3	Orf1a:T2952I,Orf1b:P314L,S:D614G
1498_InDRE_35A_M_CMX_D_15/04/2020	B.1.609	8	4	Orf1a:Y1920H,Orf1a:A4285V,Orf1b:P314L,S:D614G
5361_InDRE_27A_F_TLA_D_30/04/2020	B.1.609	8	3	Orf1a:A4285V,Orf1b:P314L,S:D614G
8279_IMSS_31A_M_CMX_D_01/04/2020	B.1	6	4	Orf1a:T265I,Orf1b:P314L,Orf3a:Q57H,S:D614G
9823_IMSS_55A_M_CMX_H_05/04/2020	B.1	6	3	N:A359S,Orf1b:P314L,S:D614G
10793_IMSS_26A_F_MEX_A_08/04/2020	B.1	8	4	Orf1a:E647G,Orf1a:S2132G,Orf1b:P314L,S:D614G
10932_IMSS_30A_M_CMX_A_08/04/2020	B.1.1	10	4	N:R203K,N:G204R,Orf1b:P314L,S:D614G
10938_IMSS_22A_M_CMX_H_08/04/2020	B.1	8	4	Orf1a:M3652T,Orf1b:P314L,Orf3a:Q57H,S:D614G
11296_IMSS_49A_M_CMX_A_07/04/2020	B.1	8	5	N:G243C,Orf1a:T4031I,Orf1b:P314L,S:D614G,S:V1122L
11398_IMSS_59A_M_GRO_D_08/04/2020	B.1.1	8	4	N:R203K,N:G204R,Orf1b:P314L,S:D614G
11433_IMSS_43A_F_BCS_A_07/04/2020	B.1.609	6	2	Orf1b:P314L,S:D614G
11461_IMSS_32A_M_GRO_A_08/04/2020	B.1	10	4	Orf1a:T50I,Orf1a:S142L,Orf1b:P314L,S:D614G
11647_IMSS_33A_F_BCS_A_08/04/2020	B.1.609	7	3	Orf1a:S4398L,Orf1b:P314L,S:D614G
11688_IMSS_48A_M_BCS_A_07/04/2020	B.1.609	6	2	Orf1b:P314L,S:D614G
11692_IMSS_50A_F_BCS_A_07/04/2020	B.1.609	6	2	Orf1b:P314L,S:D614G
11697_IMSS_37A_M_BCS_A_07/04/2020	B.1.609	7	2	Orf1b:P314L,S:D614G
11712_IMSS_41A_F_BCS_A_08/04/2020	B.1.609	6	2	Orf1b:P314L,S:D614G
11713_IMSS_35A_F_BCS_A_08/04/2020	B.1.609	6	2	Orf1b:P314L,S:D614G
11768_IMSS_42A_M_BCN_A_08/04/2020	B.1.609	9	5	Orf1a:P777S,Orf1a:P3613S,Orf1b:P314L,Orf1b:S1003G,S:D614G
11769_IMSS_24A_M_BCN_A_08/04/2020	B.1.609	8	3	Orf1a:P3613S,Orf1b:P314L,S:D614G
12060_IMSS_26A_M_MEX_A_08/04/2020	B.1	8	4	E:L73F,Orf1a:T4355I,Orf1b:P314L,S:D614G
12542_IMSS_39A_M_BCS_A_06/04/2020	B.1.609	6	2	Orf1b:P314L,S:D614G
14214_IMSS_26A_M_CMX_A_16/04/2020	B.1.1	10	5	N:R203K,N:G204R,Orf1b:P314L,Orf1b:V1706I,S:D614G
14215_IMSS_47A_F_CMX_A_16/04/2020	B.1.189	10	5	Orf1a:V2047F,Orf1b:P314L,Orf7a:E95*,Orf9b:R32L,S:D614G
16144_IMSS_27A_M_MEX_A_19/04/2020	B.1.189	9	4	Orf1a:V2047F,Orf1b:P314L,Orf9b:R32L,S:D614G
16221_IMSS_46A_M_CMX_H_17/04/2020	B.1.1	11	7	N:R203K,N:G204R,Orf1a:H712Y,Orf1a:T3058I,Orf1a:L3606F,Orf1b:P314L,S:D614G
17307_IMSS_29A_F_MEX_A_17/04/2020	B.1	7	2	Orf1b:P314L,S:D614G
17360_IMSS_38A_F_GRO_A_17/04/2020	B.1	6	2	Orf1b:P314L,S:D614G

a nt, nucleotide; aa, amino acid.

b Compared to Wuhan-Hu-1 reference genome (GenBank MN908947).

**FIG 2:**
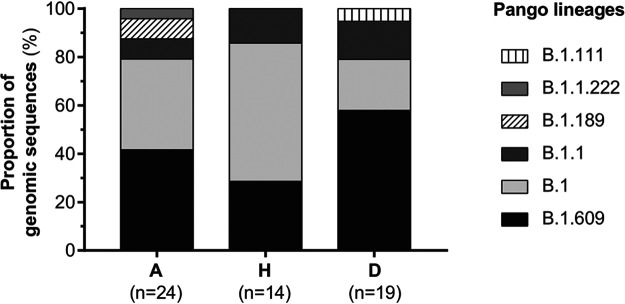
Lineage distribution across severity groups. The PANGO lineages of genomic sequences belonging to the ambulatory care (A), hospitalized (H), and deceased (D) study groups were determined using the PANGOLIN tool v3.1.7 (lineages version 2021-07-09) (54).

Phylogenetic placement revealed that the 57 Mexican isolates fell within the PANGO lineage B.1 and the descending B.1.609, B.1.1, B.1.189, B.1.1.222, and B.1.111 lineages (Table S1). None of the isolates fell within the PANGO lineage A, identified in Mexico in early March 2020 (38). The lineage distribution observed, including the replacement of lineage A over B, is consistent with reports at a global scale for that particular time (13, 39, 40). No major differences in the distribution of different lineages across the different disease outcome groups (A, H, or D) were observed (Fig. 2, chi-square test, *P* = 0.32).

**FIG 3:**
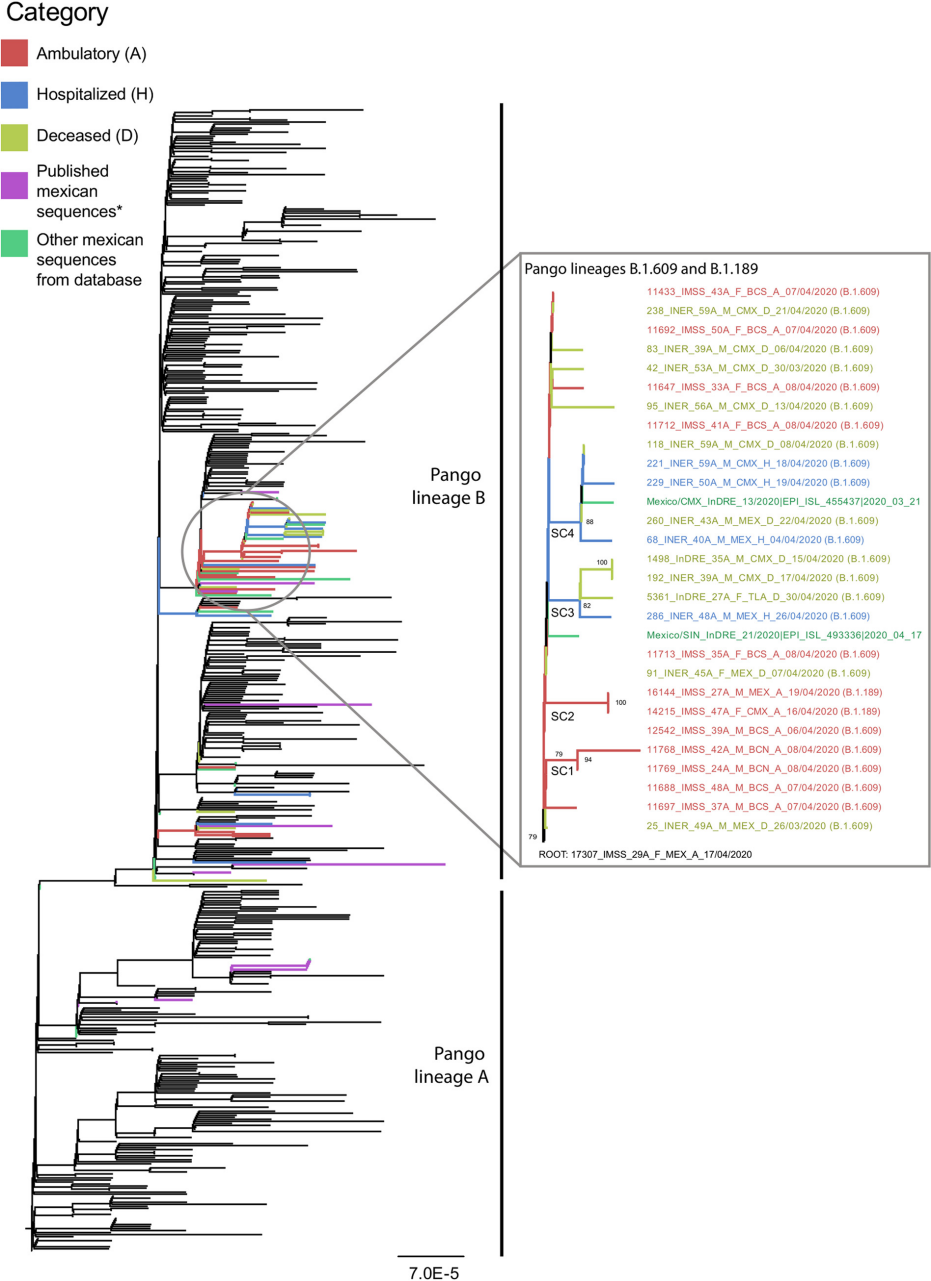
Phylogenetic placement of Mexican isolates. ML tree inferred from the Orf1ab+S alignment comprising 565 sequences, including the 57 Mexican virus genome sequences generated for this work, classified under the different disease outcome groups (A, indicated in red; H, indicated in blue; and D, indicated in yellow). An additional 34 Mexican sequences available from the GISAID platform at the time of the analysis were also included (indicated in green), together with the 17 Mexican isolates characterized from the earliest introduction events of SARS-CoV-2 within the country (indicated in purple) (26). From the 57 isolates sequenced for this study, 27 formed a single well-supported cluster belonging to the PANGO lineages B.1.609 and B.1.189. Within this single cluster, four subclusters were identified (SC1-4), all diverging from the same ancestral sequence. The 30 remaining Mexican isolates sequenced for this study displayed a scattered positioning across the tree.

Phylogenetic analysis revealed a single well-supported cluster of Mexican sequences comprising 27/57 of the isolates sequenced in this study (Fig. 3). These 27 sequences belonged to PANGO lineages B.1.609 (25 isolates) and B.1.189 (2 isolates), first detected in March and April 2020, respectively, and estimated to have originated in USA/Mexico (40). Within this single cluster, four subclusters were identified (SC1-4, Fig. 3), all diverging from the same ancestral sequence (root: 17307_IMSS_29A_F_MEX_A_17/04/2020). The 30 remaining Mexican isolates sequenced for this study displayed a scattered position across the tree (Fig. 3). Additionally, only a small cluster of sequences corresponding to the earliest isolates from the country evidencing local transmission were observed to group within the PANGO lineage A, consistent with previous observations by our group (38). Our observations suggest that most of the Mexican sequences used for this study likely represent an early but established local transmission of the virus within the country, linked to the establishment of lineages B.1.609, B.1, and B.1.1 within the region (40).

### Identification of single nucleotide variants (SNVs).

**FIG 4:**
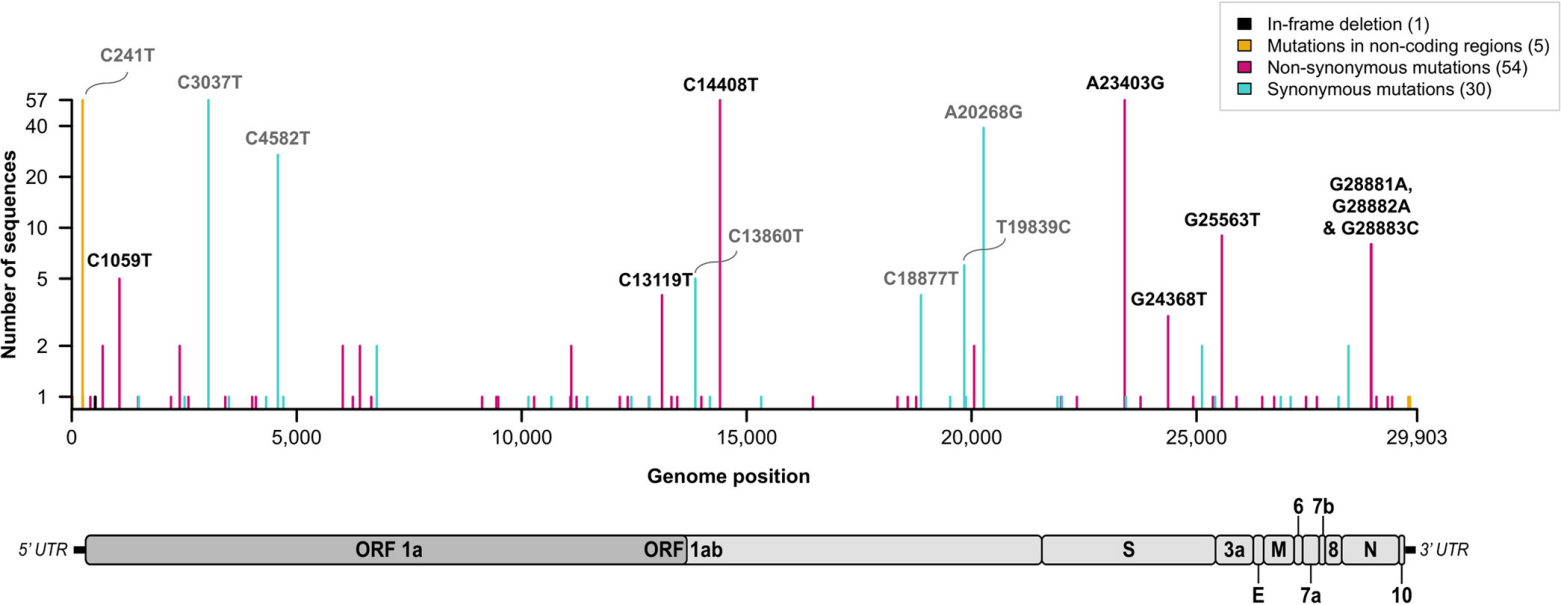
Distribution of identified mutations in the SARS-CoV-2 genome. For each single nucleotide variant identified, the number of viral isolates was plotted as a function of the nucleotide position along the SARS-CoV-2 genome. Single nucleotide variants were divided into 4 categories: in-frame deletion (black bar), mutations in noncoding regions (dark yellow bars), nonsynonymous mutations (pink bars), and synonymous mutations (turquoise bars). The number of single nucleotide variants in each category is indicated in the legend box. The SARS-CoV-2 genome organization is represented below the plot.

Mutation analysis of the 57 Mexican sequences revealed 90 single nucleotide variants (SNVs) relative to the reference genome. SNVs were mostly represented by 54 nonsynonymous mutations and in-Orf synonymous mutations, with a single in-frame deletion and a stop codon insertion detected (Table S4). Shared mutations between all Mexican sequences were observed mainly within Orf1ab and S (Fig. 4). Four nucleotide substitutions (C241T, C3037T, C14408T, A23403G), corresponding to lineage-defining mutations for PANGO lineage B (40), were present in all 57 Mexican virus genomes. The C14408T and A23403G nucleotide substitutions correspond to two non-synonymous mutations resulting in the P323L amino acid change within the viral RdRp (RNA-dependent RNA polymerase), and to the D614G amino acid change within S (spike protein). Nucleotide substitution C241T corresponds to a synonymous mutation located within the 5′ UTR (untranslated region), while C3037T corresponds to a synonymous substitution within Orf1ab/nsp3. Apart from these lineage-defining mutations, 74% of the remaining SNVs were observed within single genome sequences (Fig. 4, Table S4). Singletons observed are likely to reflect random evolutionary processes.

Amino acid changes in the spike protein have received special attention due to their potential effect on viral fitness and immune escape. In addition to the D614G substitution found in all 57 isolates, we identified 6 nonsynonymous substitutions within S (Fig. 4, Table S4), none of them located in the RBD. Two amino acid changes were detected in the N-terminal domain, three were located within the S2 subunit, and one in the cytoplasmic tail. However, these substitutions were identified in only one to three samples, precluding further analyses of their potential phenotypic impact.

### Genotype–phenotype association analysis.

Mutation analysis for the 27 Mexican isolates falling within the single cluster identified (PANGO lineages B.1.609 and B.1.189, Fig. 3) revealed six shared amino acid changes occurring across sequences: three of these within Orf1a (T265I/nsp2-85, L3606F/nsp6-38, and A4285V/nsp10-32), one within Orf1b (P314L Orf1b/RdRp-323), and two within S (D614G and D936Y) (Table S4). However, genotype–phenotype association analysis (see Materials and Methods section: “Cluster identification and genotype-phenotype association analysis”) revealed that none of these mutations appear to have a structured distribution associated with the Mexican sequences, or with the complete sampled sequence data used (Fig. S2). A potential link between the sequence subclusters and the different disease outcome groups (A, H, or D) was identified (SC1 and SC2 associated with group “A”, and SC3 and SC4 associated with groups “H” and “D”) (Fig. 3). However, as the four subclusters descend from the same ancestor node, these cannot be considered as “independent,” and any observation of genotype–phenotype associations for independent lineages is not sustained. Thus, no potential phenotype–genotype associations were detectable under a trait-association phylogenomic approach for our data set. The pattern of occurrence for shared mutations observed across the Mexican sequences suggests that most of these represent characters arising from a founder effect (41), with no apparent association with any disease outcome.

## DISCUSSION

Here we present the genomic and phylogenetic characterization of 57 SARS-CoV-2 virus genome sequences corresponding to isolates from patients with distinct disease outcomes. Our analysis revealed that genetic diversity of the characterized viruses, collected during March–April 2020 in Mexico, showed a gradual accumulation of mutations since the initial introduction of the virus until the early establishment of the epidemic in the country (38). This is shown by the local shift from the PANGO lineage A to B, and the establishment of the PANGO lineages B.1.609, B.1, and B.1.1 within the country, reflecting an extended local transmission of the virus.

We further explored any possible genotype-to-phenotype associations to detect the co-occurrence of distinct mutations with different disease outcomes. To minimize biases inherent to the host population that could impact disease severity (26), the samples used in this study were all derived from patients below 60 years of age, without any confirmed comorbidities considered to be risk factors for severe COVID-19. We found no evidence suggesting the co-occurrence of mutations associated with any particular disease outcome. Our results are consistent with previous studies from other geographical regions (28, 42), suggesting that even among individuals at low risk for severe COVID-19, the host genetic and medical background are the factors most likely determining patient clinical outcome (28). In agreement with this observation, we found that patients with mild COVID-19 (A group) were significantly younger compared to individuals belonging to the D or H severe outcome groups, in line with studies highlighting older age as a major risk factor associated with COVID-19 morbidity and mortality (26, 43).

In addition to demographic and clinical conditions, host genetic factors have also been suggested to be associated with disease severity during SARS-CoV-2 infections, increasing the risk of a severe outcome for patients who would otherwise be considered low risk based solely on demographic and/or medical condition. Some of these identified loci may be involved in regulating key host antiviral defense mechanisms or mediating inflammatory organ damage responses (such as the interferon receptor gene IFNAR2 [44]), and/or may potentially involve the ABO blood-group system (45, 46). Additional studies have shown that limited access to health care systems, and other socioeconomic determinants related to marginalization and poverty, are associated with COVID-19 severity and mortality, and may confound the observed genetic associations (26, 30, 47).

Limitations of our study include a small sample size relative to the number of cases reported within the country to the date of the analysis, resulting in a heterogeneous representation of the geographical distribution of cases across the country. Moreover, a limited genetic diversity of the virus coupled with a lack of metadata related to disease severity for other sequences from Mexico and for those globally collected, limited the exhaustive exploration of the co-occurrence of associated convergent mutations linked to different disease outcomes within a more general scenario.

Genomic surveillance programs of SARS-CoV-2 have generated an exceptionally high number of viral sequences and have allowed to monitor the evolution of SARS-CoV-2 at regional and global levels. However, many questions remain concerning the potential phenotypic effects of the identified mutations. More multidisciplinary analyses integrating genomic, epidemiological, and clinical data as well as experimental virology studies using *in vitro* and *in vivo* models are needed to further characterize the sets of mutations observed in circulating variants and their potential effects on viral fitness, virulence, and antigenicity.

## MATERIALS AND METHODS

### Ethical statement and sample collection.

The samples used in this study and their associated data are part of the national response to COVID-19, collected under Mexican Official NOM-017-SSA2-2012 for epidemiological surveillance. Clinical specimens were collected in laboratories and hospitals belonging to the Public Health Ministry of Mexico (Red Nacional de Laboratorios de Salud Publica (RNLSP); Instituto Nacional de Enfermedades Respiratorias, INER; and Instituto Mexicano del Seguro Social, IMSS). All samples were unlinked from any personal identifiers prior to commencement of the study. Oropharyngeal and/or nasopharyngeal swabs and tracheal aspirates were placed in virus transport media following official procedures for diagnostics and sequencing processing (48). Samples were collected from PCR-confirmed cases, with molecular diagnosis carried out following protocols validated by the InDRE (Instituto de Diagnóstico y Referencia Epidemiológicos, Secretaría de Salud, Mexico, National Reference Laboratory for COVID-19), as recommended by the World Health Organization (49). All samples were obtained from individuals under 60 years of age and with no diagnosed medical conditions linked to severe COVID-19 (chronic respiratory disease, cardiovascular disease, diabetes, immunosuppressive condition, and obesity class II and III).

### Sample processing, sequencing, and viral genome assembly.

Viral nucleic acid extraction, amplification, and sequencing was done as previously described (38) with slight modifications. Clinical samples that failed to render full viral genome sequences using a high-throughput approach were further sequenced under an amplicon-based protocol with the POLAR method (50) (Table S1). Sequence quality control and assembly was performed as described (38). Only full virus genome sequences with a coverage >98% and a mean depth >30× were considered for further analysis (Table S1).

### Sequence data collation.

Approximately 44,500 SARS-2-CoV-2 full genomes sampled worldwide available in the GISAID platform (4, 5) collected as of May 8th, 2020 were downloaded and aligned as part of the public data sets used for the COVID-19 Genomics UK (COG-UK) Consortium (51, 52) (Table S2). Thirty-four additional sequences from Mexico also available in the GISAID platform were included, as well as 17 Mexican virus genomes characterized from the earliest introductions of SARS-CoV-2 into the country (38). A sequence subsampling scheme from the original COG data set was done to include all sequences collected globally within the time frame of our study and up to a week after the collection date of our samples (April 30th, 2020). Subsampling was done using a random approach, since we were not looking into mutations occurring within a global context. For this, the global COG sequence alignment (excluding Mexican sequences) was randomly subsampled to 2% and sequences with the following characteristics were removed: (i) >1000-nt shorter than the full genome length, (ii) identical to each other, or (iii) containing >10% ambiguities (N or X). A total of 508 sequences retained in addition to the 57 genomes generated in this study, were used for the final data set.

### Phylogenetic placement and tree inference.

Phylogenetic placement and genetic characterization of the 57 Mexican virus genome sequences was done using the PANGOLIN tool v3.1.7 lineages version 2021-07-09 (53, 54) (Table S1). For local phylogenomic analysis, the main viral ORFs (Orf1ab and S) were extracted and aligned. Individual ORF alignments were concatenated to generate an Orf1ab+S alignment, excluding UTRs and noncoding intergenic regions. The final data set was composed of 565 sequences with a total length of 24,821 nucleotides. A maximum likelihood (ML) tree was then estimated using RA×ML v8 under a general time reversible nucleotide substitution model with gamma-distributed among-site rate variation (GTR+G) (55). Branch support was assessed using a bootstrap analysis with 100 replicates. The resulting ML tree was rooted using the Wuhan-Hu-1 reference genome. The general clustering pattern observed for the Mexican sequences was verified by comparing it to available global trees (56, 57) and to a tree generated in previous study by our group (38).

### Cluster identification and genotype–phenotype association analysis.

To search for mutations occurring at a minimum frequency of 0.01 within the sampled sequence population, variable sites across the alignment were identified by comparing across sequences and to a general consensus generated under a 95% threshold using the “Find Variations/SNPs” function in Geneious Prime v2020.0.4 (58). Sites corresponding to nonsynonymous shared mutations across the 57 Mexican sequences were coded as discrete taxon traits and subjected to ancestral state reconstruction on the ML tree under a maximum likelihood framework (RAS-ML) (59). Ancestral reconstructions were performed at a node level, representing changes occurring within nodes and not those occurring on terminal branches likely to correspond to mutations that have not been fixed in the circulating virus population.

Clusters inferred to have originated within Mexico were identified in the ML tree by detecting a minimum of two sister sequences diverging from an “ancestral” Mexican isolate, and a bootstrap support value of >50 for the joining node. Following cluster identification, the corresponding clinical metadata available for the 57 Mexican sequences were used to identify virus clusters associated with different disease outcome categories (A, D, or H groups) (Table S3, Fig. S2). Using a trait-association phylogenomic approach, the ancestral reconstruction trees were visually inspected to identify the co-occurrence of mutations (virus genotypes) within the Mexican sequence clusters, and the phenotype traits of interest (A, D, or H groups). This method allows detection of mutations that co-occur across independent sequence clusters (i.e., lineages) and that may be linked to a given phenotype (in this case disease outcome) (60–62). Any structured genotype-to-genotype association observed within the tree can be formally tested using a phylogenetically informed statistical approach (60–62), instead of using a standard χ^2^ test that does not take into consideration possible phylogenetic correlation of the sequence data.

### Data availability.

The generated sequences of SARS-CoV-2 used in this study have been publicly shared through the Global Initiative on Sharing All Influenza Data (GISAID) repository and have also been deposited in the NCBI database. Accession numbers are listed in Table S3.
